# #anatomymcq—A pilot study on using the Twitter survey tool as a formative assessment strategy

**DOI:** 10.15694/mep.2020.000141.1

**Published:** 2020-06-30

**Authors:** Sourav Bhattacharjee

**Affiliations:** 1 School of Veterinary Medicine

**Keywords:** Gross anatomy education, veterinary anatomy education, undergraduate education, veterinary students, social media usage, Twitter, Web 2.0 technology.

## Abstract

This article was migrated. The article was marked as recommended.

**Background:** Various social media portals, such as Facebook, Twitter, and YouTube, are emerging fast as effective platforms in anatomy education. Interestingly, the survey tool of Twitter can be utilized to develop multiple-choice questions, where the students may cast their votes in favor of the correct option. To assess the utility of the tool, a study was undertaken over fourteen weeks (twelve weeks of tutorials and two weeks of study break before the end-of-term examination) during the second semester of the 2016-2017 academic year in the University College Dublin School of Veterinary Medicine under voluntary and anonymous participation.

**Methods:** One survey with four alternatives and marked with #anatomymcq was posted on each day of the week, while the correct answer was revealed the following day, also marked with the #anatomymcq-thus, a total of 67 such surveys broadly classifiable under the following topics: gastrointestinal system (n=14), urinary system (n=12), comparative anatomy (n=10), embryology (n=10), reproductive system (n=14) and applied anatomy (n=7) were tweeted.

**Results:** Altogether 263 votes were received (highest in the weeks 7 and 8) for the 67 surveys in the following order: GI system (6.50 votes/survey) > urinary system (4.33 votes/survey) > comparative anatomy (3.60 votes/survey) > embryology (2.90 votes/survey) > reproductive system (2.64 votes/survey) > applied anatomy (2.57 votes/survey). The average daily impressions for the surveys were significantly ( *p*<0.05) higher than the first week, while the weekly mean engagement rate grew from the first week as well. The weekly total number of hashtag clicks also increased. The student feedback was positive on the exercise.

**Conclusions:** The integrated data highlight the potential of such a tool while identifying its strengths and weaknesses and was able to provide an interactive tool to engage students outside the formal teaching hours while facilitating student learning and engagement.

## Introduction

The scope of anatomy pedagogy and its subsequent struggle to adapt within the landscape of the medical curriculum-both in human and veterinary medicine, is transforming fast. Introduction of various technological advancements along with the rise of social media infinitely connecting the minds and people across the globe are impacting human lives, and so the field of higher education, including medical scholarship ( Jalali *et al.*, 2013, 2015; Carroll, Bruno and vonTschudi, 2016). The reach of social media, e.g., Twitter (Twitter Inc.; San Francisco, CA, USA), Facebook (Facebook Inc., Menlo Park, CA, USA), LinkedIn (LinkedIn Corp., Sunnyvale, CA, USA), YouTube (YouTube LLC, San Bruno, CA, USA), especially within the younger generation often termed as the *Net Generation* or *Millennials*, is undeniable; which despite its weaknesses (e.g., addiction, lack of monitoring, and circulation of wrong information, both unintentional and deliberate) also opens up avenues for innovative, competent and credible methodologies in anatomy education ( Buzzetto-More, 2012; Pant *et al.*, 2012; Cheston, Flickinger and Chisolm, 2013; El Bialy and Jalali, 2015; Barry *et al.*, 2016; Roy *et al.*, 2016; Stephens and Gunther, 2016; Sterling *et al.*, 2017; Chytas, 2018). The scope of such emerging digital platforms are being magnified further due to the practical constraints associated to the growing class sizes, limited resources including trained personnel or funding, and increasing burden of the syllabus to be covered within the reduced teaching hours-as noticed in multiple countries, such as the USA, UK and Australia ( Heylings, 2002; Drake *et al.*, 2009; Craig *et al.*, 2010). Such challenges are further augmented with the inherent difficulty and complexity ingrained to anatomy as a discipline, which, albeit being an essential skill to practice medicine with competence ( Smith and Mathias, 2010), is also known to cause stress and lack of engagement in medical graduates.

**Figure 1.**
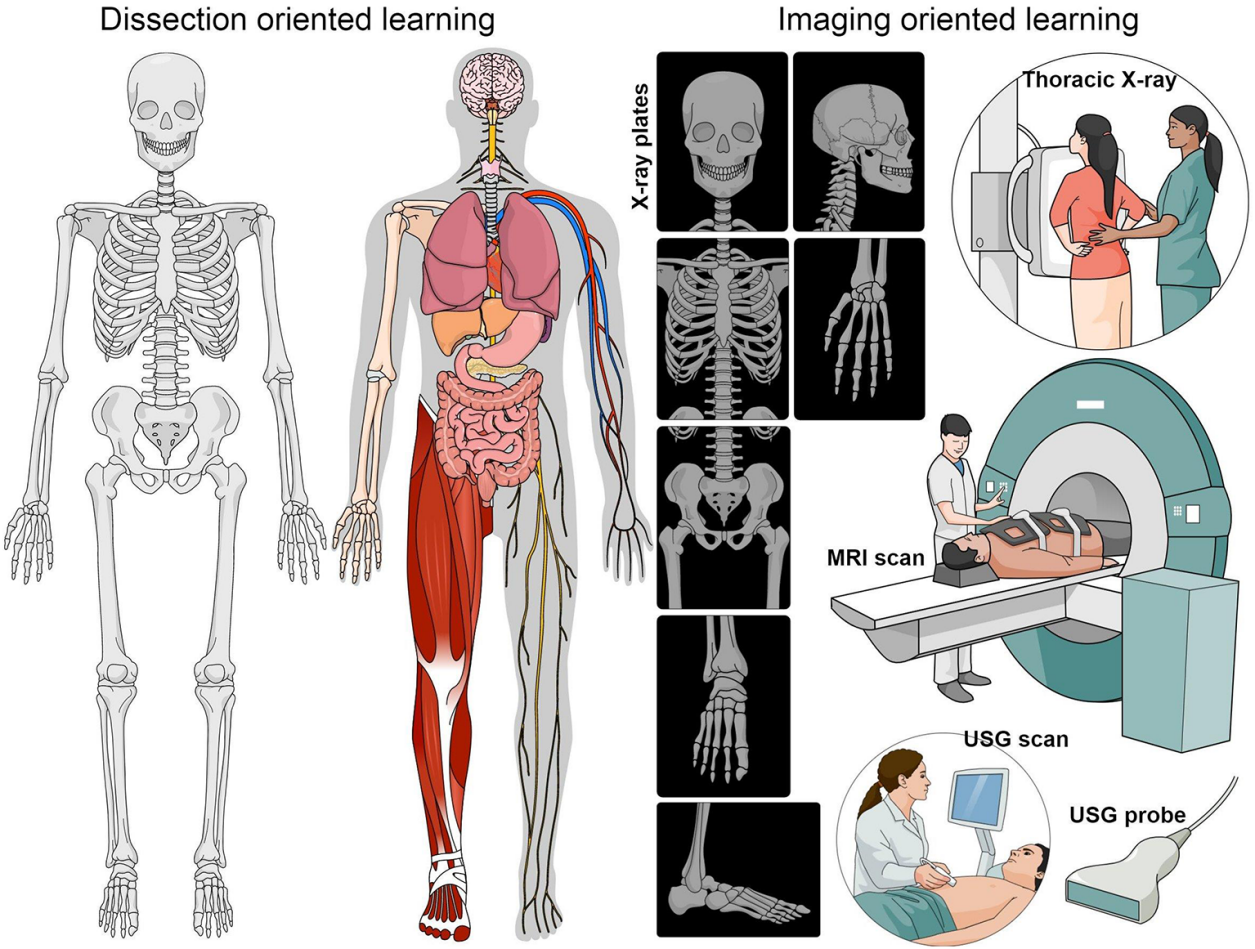
Cartoon showing the various conventional ways of anatomy teaching practiced within the current medical curriculum, including dissection and imaging-oriented learning modalities, such as the CT and USG scans.

Formative assessment is an essential component of medical education and had been reported to enhance the learning experiences of students along with a better understanding of the subjects and preparation for summative assessments ( Rolfe and McPherson, 1995; Black and William, 1998; Bierer *et al.*, 2008; Carrillo-de-la-Pena *et al.*, 2009; Evans, Zeun and Stanier, 2014). It is known that when offered systematically, under non-penalizing circumstances, and aided with timely feedbacks, a well-designed formative assessment strategy can help students in identifying the gaps in their knowledge, familiarize themselves with the type of questions to be asked during summative assessments, and enhance active participation ( Harlen and James, 1997; Krasne *et al.*, 2006). The term social media, abbreviated often as SoMe, has frequently been used as an umbrella term for a wide range of online applications including networking, multimedia hosting, and sharing of written pieces, such as blogging or news feeds in forums ( McKenna and D′Alessandro, 2011; Nadkarni and Hofmann, 2012; Meshi, Tamir and Heekeren, 2015). Such social media portals present with an unorthodox tool with the potential of addressing many of the existing challenges in medical education ( Hennessy *et al.*, 2016). With its inbuilt visual representation, arresting infographics, instant accessibility aided by the explosive growth of users of handheld devices ( Ozdalga, Ozdalga and Ahuja, 2012; Baran, 2014; Gökçearslan Ş *et al.*, 2016), it is evident that such platforms have the potential to revolutionize the teaching and learning in anatomy ( Figure 1), such as cadaveric dissection, an inspection of specimens and prosected materials in conjunction to imaging, such as the X-ray, computed tomography (CT), magnetic resonance imaging (MRI) scans or ultrasonography (USG).

Use of social media platforms as addenda to the theoretical lectures, especially during the dissection classes or in-depth discussions on the intricate details may impact the current anatomy education to remain relevant and yet, able to generate curiosity among the millennials used to having information-at will, and at ease ( Roberts, 2005; Skiba, 2008; DiLullo, McGee and Kriebel, 2011). Maintaining a sustained stream of connectivity along with a steady supply of information while developing social groups was identified to facilitate the engagement of students and nurture the way they interact or collaborate. Naturally, various technology-enhanced learning (TEL) tools including the podcasts or development of online platforms, e.g., Blackboard (Blackboard Inc., Washington D.C., USA) were initially recommended ( Cecot, Smith and Border, 2014; Pickering, 2015); although with rare exceptions of engagement rates as high as ≥90% ( Junco, Heiberger and Loken, 2011; Junco, Elavsky and Heiberger, 2013), the engagement of students remained ~24 %, especially while conducting online forums ( Oliver and Shaw, 2003; Jayakody *et al.*, 2014). Similarly, the scope of LinkedIn is limited, given it is mostly used by the established professionals for networking purposes, while YouTube is primarily a site for hosting videos. In contrary, students were found to interact more on free social media portals, such as the Facebook, which provides a sense of community, while at the same time offers a space to collaborate, contribute, and comment on the issues of relevance ( Jaffar, 2012; Hall, Hanna and Huey, 2013; Foley, Maher and Corrigan, 2014; Mukhopadhyay, Kruger and Tennant, 2014; Jaffar *et al.*, 2016; Pickering and Bickerdike, 2017). However, students did not encourage the presence of teachers in the same Facebook pages, perhaps due to the feeling of an intrusion into privacy, and awkwardness ( Jaffar, 2014).

Established in 2006 and currently with ~350 million registered users, Twitter as a Web 2.0 technology ( Kassens-Noor, 2012; Jacquemin, Smelser and Bernot, 2014; Kimmons and Veletsianos, 2016), has emerged as one of the most popular microblogging platforms where the registered users can send messages *via* short message service (SMS) or retweet posts from others ( Thames, 2009; Bista, 2015). It allows the users to open multiple accounts conducive for the students to maintain separate accounts for studies and private communications-thus, maintaining a distance between their professional and personal spaces ( Ebner *et al.*, 2010; Schroeder, Minocha and Schneider, 2010; Forgie, Duff and Ross, 2013). The tweets were initially allowed to be only ≤140 characters long; however, such length restriction was relaxed to 280 characters from the 7 ^th^ November 2017. In 2015, Twitter introduced the option of posting surveys as tweets where a maximum of four options could be placed for online polling and enabled the users to seek the opinion of the vast audience pool in Twitter through anonymously cast votes. The end-results of the surveys show the total number of votes received, and the percentage of total votes registered in favor of each option.

Interestingly, such Twitter-based surveys can be utilized to create multiple-choice questions (MCQs) and can be a part of the formative assessment strategy within anatomy education. Previously, there had been sporadic attempts to explore the potential of Twitter in enhancing the anatomy learning experience ( Lin, Hoffman and Borengasser, 2013; Carpenter and Krutka, 2014; Hennessy and Border, 2015; Tur and Marn, 2015; Ricoy and Feliz, 2016; Veletsianos and Kimmons, 2016). Some previous initiatives used the Twitter survey tools for posting anatomy MCQs in similar fashion by posting a volley of MCQs as surveys on the same day, and although a commendable initiative, did not engage the students systematically over a sustained period, as in this study. As an anatomy teacher and within the capacity of being the coordinator of two modules on veterinary abdominal and pelvic anatomy, the author conducted a study for fourteen weeks (23/01/2017-12/05/2017 including two study weeks before the end-of-term examination and excluding the two weeks of mid-term break) during the second semester of the 2016-2017 academic year using his Twitter handle, where surveys were tweeted daily as practice MCQs.

The objectives of the study were: (i) to utilize the Twitter survey tool as a platform for developing MCQs and offer them to the students for practice; (ii) to run a study over the entire semester while providing daily MCQs that may establish a resource for the students to learn and engage during out of the regular teaching hours; (iii) to prepare the students on the type of MCQs to be asked at the summative assessments; (iv) to improve the skills of framing MCQs within limited words or characters; and finally, (v) to gain experience as an anatomy educator on the strengths and weaknesses of using Twitter survey tool for such purposes. The hypothesis was that such a platform would provide the students with a valuable resource for learning, while the expectation was that such emerging SoMe platforms would be able to develop into an interesting, fun and interactive platform for MCQ practice, which would energize the preparation before dissection, nurture collaboration, and facilitate the preparation for the end-of-term assessments.

This account summarizes the experiences of an anatomist while interacting with the students through such an emerging platform and prioritizes its salient features, which, apart from spotting its vulnerabilities, will also be able to distill out the utility of such tools when used strategically. Furthermore, the ambition of this report is to reach out to the broader community of anatomy educators interested in exploring the prospects of such emerging tools and present a balanced critique in order to make them aware of the strengths and weaknesses of the entire exercise.

## Methods

### Study design

**Figure 2.**
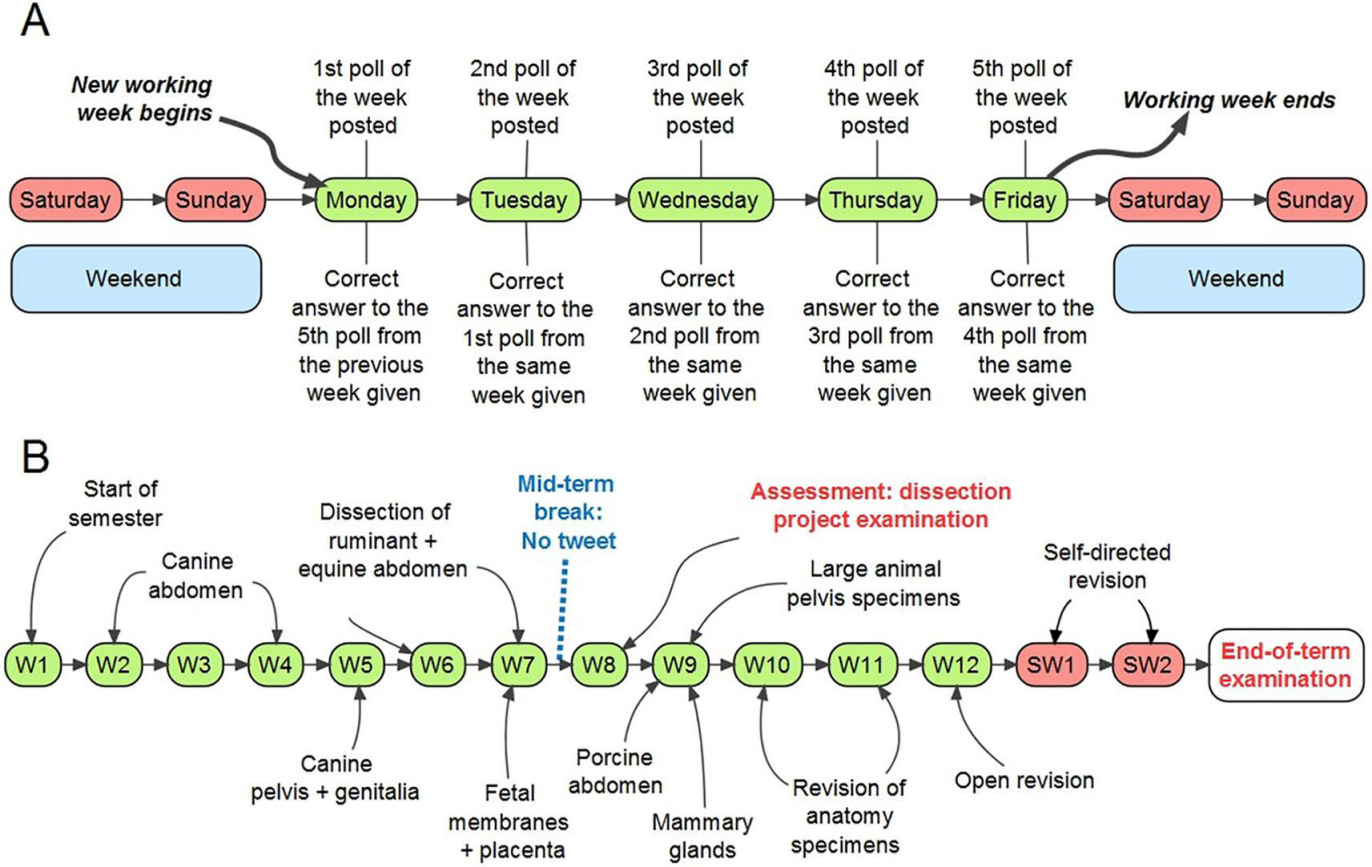
(A) Scheme showing the weekly study design with the schedule of posting the Twitter surveys and collection of data after that, (B) scheme showing the organization of the modules through the fourteen weeks-long semester with subjects taught along with the schedule of the revision classes and summative assessments [Abbreviations: W, week; SW, study week].

An ethical exemption was obtained from the research ethics committee, given the fully anonymous nature and voluntary participation of the audience. The study was conducted within the fourteen weeks-long second semester of the 2016-2017 educational year (23/01/2017-12/05/2017), while the end-of-term examinations were conducted on the late May 2017 with two weeks of study break preceding the examinations. The Twitter surveys, designed in alignment with the learning objectives of the modules (Supplementary File 1), were tagged with #anatomymcq to make them easy to find, follow, or interact. Each survey had four options framed within the permissible 25 characters, while only one option denoted the correct answer. A survey was posted daily while the correct answers, also tagged with #anatomymcq, were tweeted the next day, roughly after 24 hours, along with the new survey ( Figure 2A). The surveys were tweeted on each working day of the week, that is, Monday-Friday and were open for 24 hours; except the ones posted on the Fridays which were left open till the following Mondays, that is, for 72 hours to cover the weekends (Saturdays and Sundays). In case of bank holidays (Good Friday on 14/04/2017 and Easter Monday on 17/04/2017), the survey posted on the preceding Thursday (13/04/2017) was left open till the forthcoming Tuesday (18/04/2017), that is, for five days. Extra practice surveys as revision materials were posted during the two study weeks. Thus, altogether sixty-seven such surveys-four on the first week followed by five-per-week for the next 11 weeks (excluding the Good Friday and Easter Monday) in addition to the five-per-week for the two study weeks, were tweeted. In alignment to the ongoing modules, all the sixty-seven surveys were selected from the abdominal and pelvic anatomy, and were broadly categorized under the following six topics: (1) *Embryology* (n=10) covering the development of the abdominal and pelvic organs including the rotation of gut and organogenesis; (2) *Gastrointestinal (GI) system* (n=14) covering the gross anatomy of the gut including the hepatopancreatobiliary axis along with blood supply and innervation; (3) *Urinary system* (n=12) including the kidneys, adrenal glands, ureters, urinary bladder and urethra; (4) *Reproductive system* (n=14) including the anatomy of gonads, penile anatomy with the erectile mechanism, and the bony pelvis along with its four diameters (vertical, transverse, oblique, and conjugate in canine anatomy); (5) *Comparative anatomy* (n=10) covering the species variations found in the various farm animals e.g., horse, ruminants (cow, sheep), and pigs; and, (6) *Applied anatomy* (n=7) topics, such as the inguinal and femoral canals, muscles of the abdominal wall (external and internal obliques, transversus abdominis and rectus abdominis muscles, rectus sheath), various regions of the abdomen (xiphoid, hypochondriac, umbilical, lateral, inguinal and pelvic in canine anatomy), abdominal incisions (ventral midline, paramedian, paracostal, flank), and spine. The entire collection of the surveys, classified under the six topics, are provided as Supplementary File 2. The two modules coordinated by the author had separate groups of students: an undergraduate-entry group of 85 students, and a graduate-entry group comprising of 30 students (average age 22-25) with a graduate degree-thus, the entire cohort comprised of 115 students. The graduate- and undergraduate-entry groups conducted dissection on cadavers on Mondays and Thursdays, respectively, while the summative assessments ( Figure 2B) included a dissection project examination conducted on the eighth week of the term followed by an end-of-term examination. The students were made aware of the study in the first week.

### Collection of feedback from students

A standard, voluntary and fully anonymized online feedback form was made available to all the students registered for the two modules by the institute’s Blackboard (Bb) system where the following three questions were included: (i) the assessment was relevant to the work of the module, (ii) I achieved the learning outcomes for this module, and, (iii) the teaching on this module supported my learning. The students were asked to consider their experiences regarding the Twitter-based surveys while responding to the feedback collection form. The responses were collected in a 5-point Likert scale (strongly disagree, disagree, not sure, agree and strongly agree), while the ratings were converted into a point-based score: 1=strongly disagree; 2=disagree; 3=not sure; 4=agree; 5=strongly agree with calculation of the mean and standard deviation (SD). Additionally, the author himself interacted with the students on multiple occasions to collect their opinions and thoughts on such an exercise.

### Data collection and statistical analyses

After the study, the following numerical data were extracted using the *Tweet Activity* wizard: number of impressions, hashtag clicks (as an indication of growing interest on the tweeted surveys), and the total number of engagements including the number of cast votes for each of the surveys. The numerical data, along with the overall weekly statistics, were exported to Microsoft Excel (Microsoft Corporation, Redmond, WA, USA) spreadsheets for each survey. The percentage engagement rate for each survey was calculated by the following equation:

Engagement rate (%) = (Total number of engagements / Total number of impressions) x 100

Here, the term *impression* referred to the number of times the survey showed up in someone’s Twitter timeline, while the term *engagement* represented a collection of the following activities: casting votes, retweets, replies, follows, favorites, links, cards, hashtags, embedded media, username, profile photo, and tweet expansion. The obtained data were analyzed and plotted as mean ± standard deviation (SD) using the OriginPro ^®^ 2015 data analysis and graphing software (OriginLab Corp., Northampton, MA). A double-tailed Student’s *t*-test was performed, and significantly different data points ( *p*<0.05) compared to the reference data-as mentioned in the figure legends, were marked with an asterisk (*) symbol.

## Results/Analysis

### The trend of voting over the weeks

**Figure 3.**
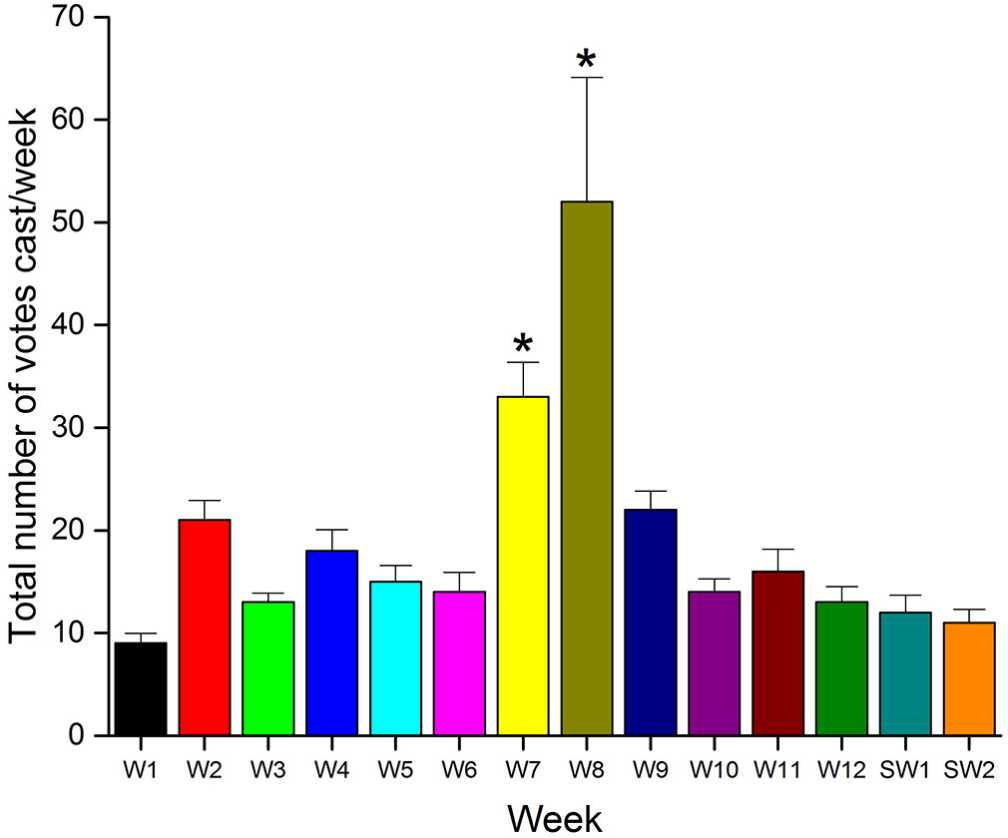
The total number of votes cast per week, while the data for each week is shown as mean ± SD (n=4 for the weeks 1, 10, and 11; while n=5 for all the remaining eleven weeks). The distribution of data points for each of the weeks was compared to the first week and significantly different (p<0.05) data points on the weeks seven and eight were marked with an asterisk (*) symbol [Abbreviations: W, week; SW, study week].

Altogether 263 votes were cast for the total 67 surveys with the lowest and highest number of votes received for an individual survey being 1 and 32, respectively. The weekly pattern of voting was heterogeneous and varied across the length of the study-from 9 in week 1 to the highest 52 votes in week eight ( Figure 3). Only weeks 7 and 8 were significantly different ( *p*<0.05) from the first week with *p*-values of 0.042 and 0.046, respectively ( Table 1).

**Table 1.** The weekly data on audience interactions noted for the Twitter surveys

Week no.	Total number of impressions	Total number of engagements	Total number of votes	Total number of # clicks	Weekly ER (%)
Week 1	217	29	9	5	13.36
Week 2	370	68	21	17	18.38
Week 3	393	66	13	37	16.79
Week 4	554	208	18	60	37.55
Week 5	544	196	15	90	36.03
Week 6	754	242	14	115	32.10
Week 7	1010	289	33	151	28.61
Week 8	1157	341	52	135	29.47
Week 9	1312	286	22	136	21.80
Week 10	1055	238	14	111	22.56
Week 11	978	160	16	86	16.36
Week 12	1027	299	13	112	29.11
Study week 1	983	277	12	119	28.18
Study week 2	967	288	11	156	29.78

[Abbreviations: ER, engagement rate]

### The trends of voting over the topics

**Figure 4.**
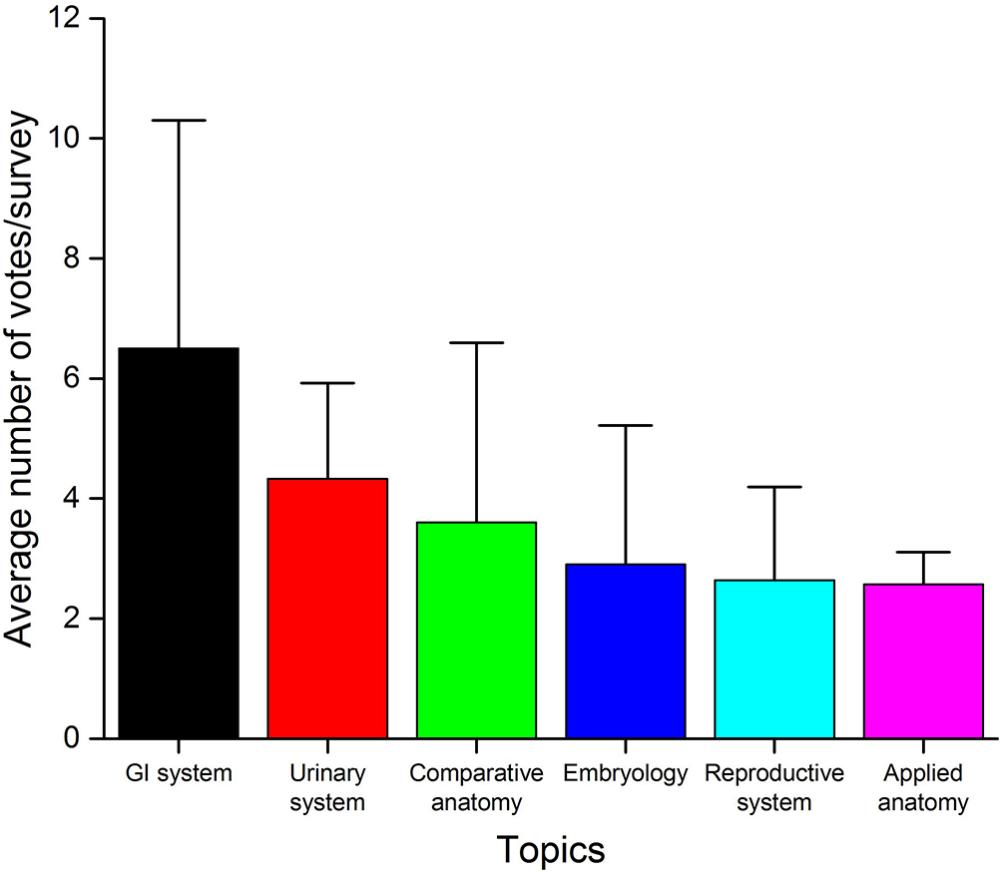
The average number of votes received per topic over the entire study period. Results are shown as mean ± SD (n=14 for the GI system, n=12 for the urinary system, n=10 for comparative anatomy, n=10 for embryology, n=14 for reproductive system, and n=7 for applied anatomy). The GI system received the highest number of votes per survey, whereas the least number of votes per survey were noted for the reproductive system and applied anatomy topics.

The average number of votes received by each of the six major topics ( Figure 4) were as follows: GI system (6.50 votes/survey) > urinary system (4.33 votes/survey) > comparative anatomy (3.60 votes/survey) > embryology (2.90 votes/survey) > reproductive system (2.64 votes/survey) > applied anatomy (2.57 votes/survey).

### Number of weekly average daily impressions and engagement rate

**Figure 5.**
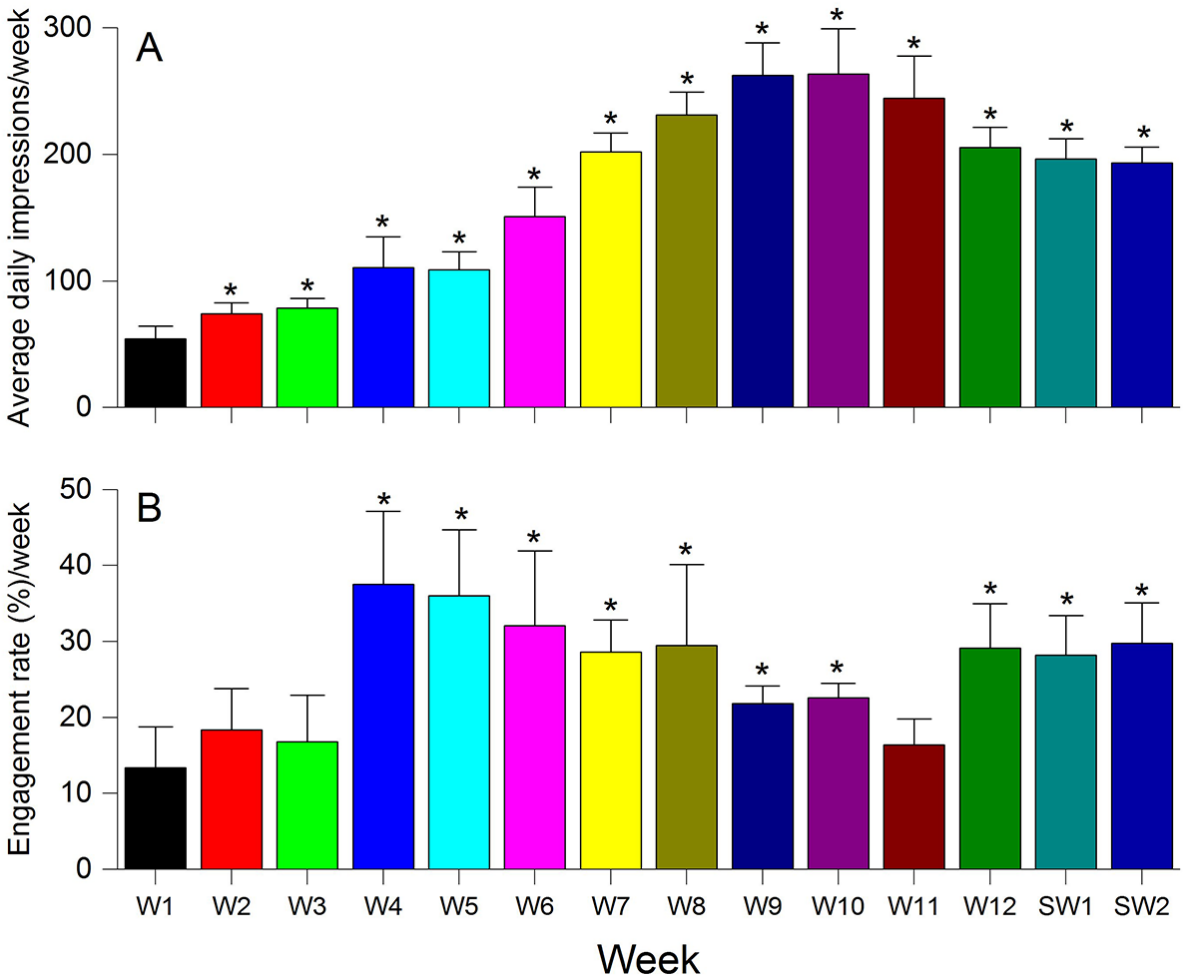
(A) The weekly total number of impressions obtained, and the data for each week is represented as mean ± SD (n=4 for weeks 1, 10, and 11; while n=5 for all the remaining eleven weeks). The distribution of data points for each of the weeks was compared to the first week, and significantly different (p<0.05) data points were marked with an asterisk (*) symbol. (B) The weekly ER (engagement rate) expressed as a percentage, and the data for each week is represented as mean ± SD (n=4 for the weeks 1, 10, and 11; while n=5 for all the remaining eleven weeks). The distribution of data points for each of the weeks was compared to the first week, and significantly different (p<0.05) data points were marked with an asterisk (*) symbol [Abbreviations: W, week; SW, study week].

After a slow start (217 in the week 1), the daily average impressions/week ( Figure 5A) grew steadily through the twelve weeks of teaching (1027 in week 12), and the trend was overall sustained over the two study weeks (983 and 967 in the first and second study weeks, respectively). All the other weeks, that is, weeks 2-12, and both the study weeks were significantly different ( *p*<0.05) compared to the first week. The weekly engagement rate ( Figure 5B) also showed an increase; 13.36% in week 1 that gradually increased to 29.11% in week 12-although the growth was less remarkable than the total number of impressions. The weekly engagement rates for the two study weeks were 28.18% and 29.78%. Weeks 4-10, 12, and both the study weeks were significantly different ( *p*<0.05) compared to the first week.

### Weekly number of hashtag clicks

**Figure 6.**
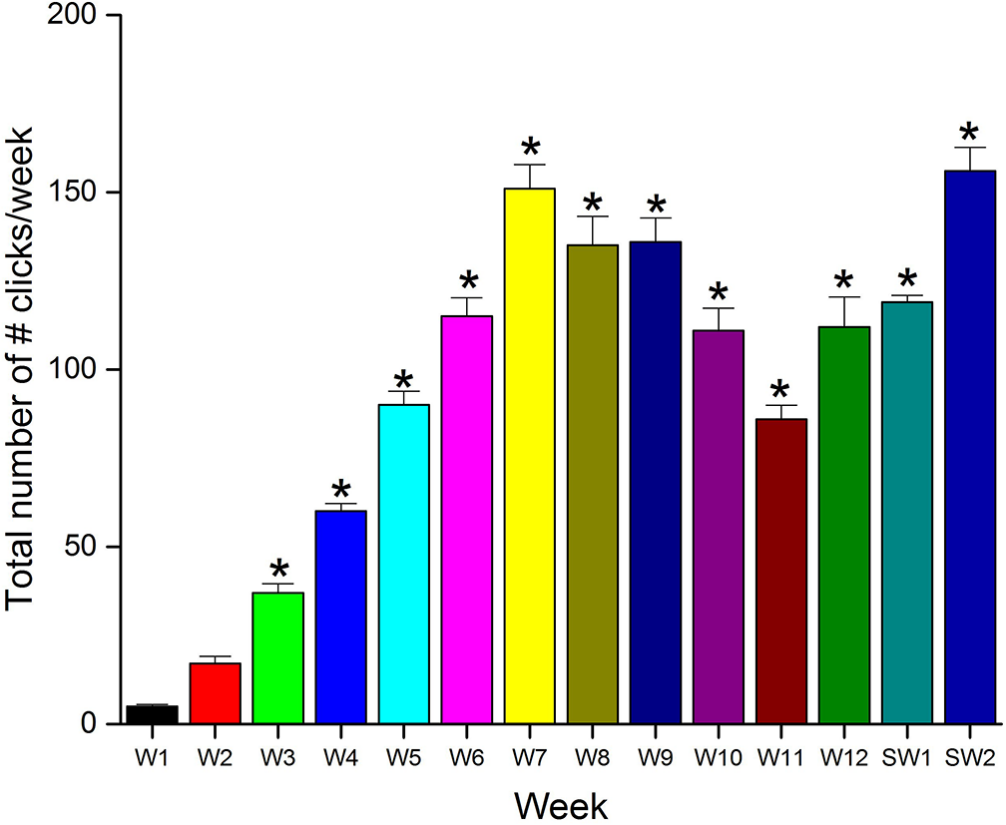
The weekly total number of hashtag (#anatomymcq) clicks and the data for each week is represented as mean ± SD (n=4 for weeks 1, 10, and 11; while n=5 for all the remaining eleven weeks). The distribution of data points for each of the weeks was compared to the first week, and significantly different (p<0.05) data points were marked with an asterisk (*) symbol [Abbreviations: W, week; SW, study week].

The weekly total number of hashtag clicks on #anatomymcq increased throughout the study-from 5 in week 1 to 112 in week 12 ( Figure 6) followed by an expected increase over the study weeks due to the ongoing preparation by the students for the summative end-of-term examination: 119 and 156 in the study weeks 1 and 2, respectively. Weeks 3-12, and both the study weeks were significantly different ( *p*<0.05) compared to the first week.

### Student feedback

**Figure 7.**
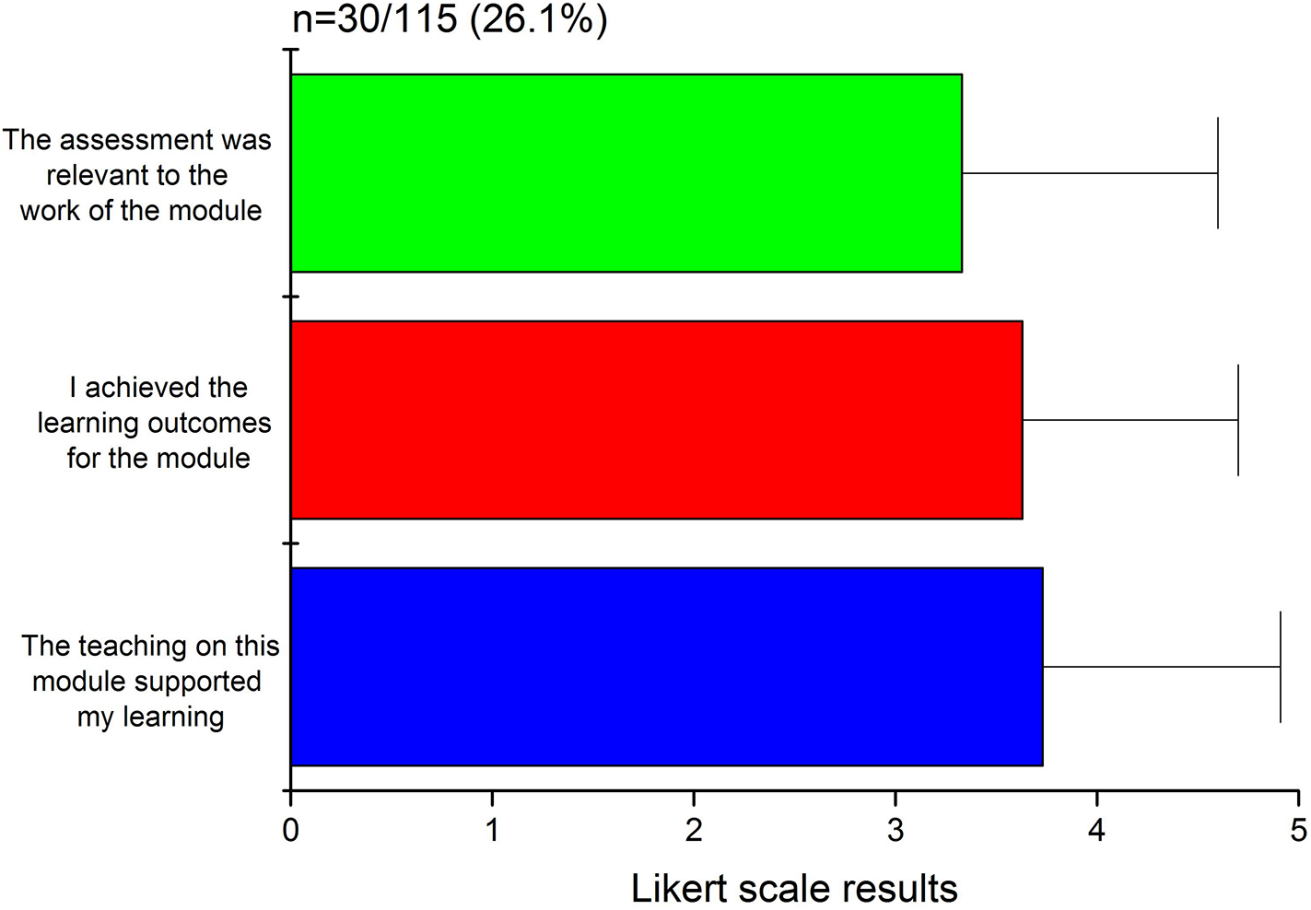
The student feedback expressed on a five-point Likert scale (1=strongly disagree; 2=disagree; 3=not sure; 4=agree; 5=strongly agree) as mean ± SD (n=30), while the corresponding queries are mentioned along the axis of the bar chart.

The student feedback received *via* the online, anonymous and standard institutional student feedback collection system for the three following questions: (i) the assessment was relevant to the work of the module, (ii) I achieved the learning outcomes for this module, and, (iii) the teaching on this module supported my learning, generated 26.1% response rate with average Likert scale scores (mean ± SD) of 3.73 ± 1.18, 3.63 ± 1.07 and 3.33 ± 1.27, respectively ( Figure 7). The verbal feedback obtained from the students on multiple occasions was overall positive, with the students praising the usefulness of such an interactive tool that nurtured collaboration, provided engagement during out of the regular teaching hours, and offered a decent resource of MCQs before the end-of-term examination. However, the students opined that the range of topics covered by the surveys could have been broader and more focused, while required better integration within the inventory of available resources.

## Discussion

An estimated one billion people use various social media platforms each month, making its impact felt across the globe. These platforms together present with an ensemble tool as visual learning environments (VLEs) or learning management systems (LMSs) with possibilities to enhance learning experiences of the medical graduates ( Veletsianos, Kimmons and French, 2013) in alignment to the various theories of learning, e.g., social constructivism, constructivism and social cognitive learning ( Bandura, 1971, 1986, 2001; Vygotsky, 1978; Wenger, 2000; Aydin, 2012; McAndrew and Johnston, 2012; Cartledge, Miller and Phillips, 2013; Lafferty and Manca, 2015; Asiri and Househ, 2016; Kakushi and Évora, 2016; Whyte and Hennessy, 2017). It is established that such social media-based teaching can facilitate learning in multiple ways, such as active learning, increased collaboration, growing engagement, self-education, opportunities for informal and incidental learning, and development of common ground between the formal and informal learning modalities ( Ajjan and Hartshorne, 2008; Tess, 2013; Ali, 2016; Guraya, 2016). It is worth noting here that as a teaching tool, social media is a new arrival to the scene ( Booth and Hultén, 2003; Boyd and Ellison, 2007; Mazer, Murphy and Simonds, 2007), and as the initial excitement began settling down, caution had been voiced by some educators on its transformation into a credible teaching tool ( Selwyn, 2010; Roblyer *et al.*, 2010; Shelton, 2017).

Overall, the voting pattern throughout the fourteen weeks was heterogeneous and lacked a specific pattern. However, compared to the other topics, it was evident that the interactions were lesser for the surveys from the reproductive system. Students have struggled to cope with the concepts of pelvic anatomy, which is understandable given its immense complexity. Additionally, the pelvic anatomy, including the reproductive system, was discussed from fifth week onward in the semester, which permitted lesser time for the students to engage with the topic. On the contrary, the GI system showed a decent engagement, which might be due to a combination of its lesser complexity, ease of understanding, and availability of more time. One of the surveys from the GI system posted on the 27/03/2017 (Monday) showed an unexpected spike in the number of total votes, that is, 32-which might have also skewed the data on an average number of votes registered for the GI system. The average number of votes cast as well as the number of impressions, number of #anatomymcq clicks, and weekly engagement rates picked up gradually throughout the study. However, the small size of the cohorts might have been restrictive in obtaining a higher response, which indicated growing visibility and audience reach as the study progressed-may be catalyzed by the word-of-mouth publicity to provide the noted momentum. The engagement rate sometimes reached 30%, which tallied well with the overall response rate of 26.1% noted in the standard student feedback collection system. It was ~50% higher than the average response rate recorded for the other modules in the institute.

The survey tool of Twitter was an effective strategy to engage students and provided ways to quantify the audience interactions in the form of numerical readouts. Twitter is a free-to-use platform, and on multiple occasions, the students informed that they liked the approach due to its ease of interaction and accessibility. Moreover, once the votes were cast, the results of the poll were displayed instantly. Therefore, one could check the vote share in favor of each choice immediately after voting and could investigate further whether the popular opinion reflected the correct answers. However, sometimes the number of votes was too low to draw firm inferences. Still, the study provided enough background material that will be helpful while using Twitter in more effective ways in anatomy education, particularly while dealing with a larger cohort. During verbal as well as written communications with the students, the author sensed their enthusiasm, which, in a way, provided them with an interactive and flexible platform to engage. Some of them noted that the variety of topics covered in the surveys could have been wider, and the number of surveys provided each day could be increased from one to three or four, enabling the inclusion of more topics. However, it was evident that the full potential of such a platform can only be realized upon careful integration into the curriculum in alignment with the learning objectives. Unfortunately, a concrete understanding or an overall consensus is currently lacking on how and when to administer such digital inputs within the curriculum, which is an issue that needs to be addressed to avert the risks of having too many unsupervised platforms providing a diverse set of assessments.

As an anatomy teacher, it was a good practice toward gaining skills in designing MCQs with minimal wording. Within the evolving landscape of academia, where outreach activities are becoming more important, it can be an interesting exercise for the faculty members for profile building and stay connected to both peers and students. The author did notice a 37% increase in the rate of gaining followers during the study period, and the growing presence within the virtual world due to this sustained tweeting can explain this temporary surge. As a teaching tool, the Twitter surveys harbor potential indeed, however, unless the current challenges are addressed, its scope will be largely restricted to formative assessments only. So far, the critical evaluation of the utility of Twitter in anatomy education had oscillated both in favor ( Rheingold, 2010; Corbeil and Corbeil, 2011), and against the notion ( Wankel, 2009). The author is convinced that such a platform will not emerge as a disruptive educational technology in the immediate future. Still, it certainly has utility in anatomy teaching, particularly as a supplement to the traditional methods of teaching, while useful in stimulating class-wide discussions and endorse critical reflection, collaboration, knowledge-sharing, or communications between the students ( Cherney, 2008). It is even more relevant in contemporary higher education, where students now prefer multimodal teaching platforms to cater to their ways of learning. An institutional policy with clarity on guidelines toward fair, responsible, and ethical use of social media in higher education, particularly within the backdrop of growing concerns over privacy, is required before such virtual tools can be included as teaching platforms. With the evolution of VLEs used widely in the western medical schools now, such as Blackboard or Brightspace (D2L, Kitchener, Ontario, Canada), it is possible to provide such practice MCQs with similar data analytics in an equally strategic, timely, and integrated manner.

### Study limitations

Conducting such surveys over a sustained period demanded time commitment that may be difficult for a faculty to offer. Unlike regular tweets or the Facebook-based surveys, the Twitter surveys, unfortunately, could not be scheduled in advance, and each survey had to be tweeted live. The author had to make sure that the surveys were tweeted daily, almost at the same time in synchrony to the correct answers posted the next day. It was quite tedious to sustain the routine over extended periods-something for consideration in the future. During the study, that is, early 2017, only 140 characters were allowed for each survey, which was often not enough to write the MCQ-stems. Additionally, only 25 characters were allowed for each of the four options, which was also inadequate sometimes. Fortunately, this issue has mostly been resolved now due to the relaxation of such restrictions. However, the 25-character restriction for writing the options, unfortunately, remains as of now (April 2020). Unlike the regular tweets, images were not allowed to be embedded within the surveys-thus, MCQs based on the images of prosected materials or specimens could not be asked ( Holland, OʹSullivan and Arnett, 2015). It is a drawback of the Twitter-based surveys when compared to the flexibility offered by Facebook that allows the surveyor to write the stems in unlimited characters, or have more than four options (if required); and most importantly, to embed figures. Another limitation of the study was the inability to correlate such Twitter interactions to individual performances due to anonymity. Furthermore, the surveys could only be accessed *via* a valid Twitter account-thus, not available to students absent on Twitter. Such anonymity also made it challenging to prevent contamination from external users.

## Conclusion

In summary, a study was undertaken over fourteen weeks while tweeting a survey daily as mock MCQs to explore its utility as a formative assessment strategy. The data suggested growing interest and student engagement with the surveys, albeit lacked some of the tools required for proper assessment of its efficacy (e.g., correlating student engagement to individual student performance) due to the anonymous nature of the study. The available numerical read-outs extracted *via* the inbuilt data analytics suite indicate that if used rationally and timely, such tools can facilitate the learning experience of students by providing an interactive platform able to maintain touch with the students beyond the usual teaching hours. However, proper integration of such tools within the curriculum remains a challenge that needs to be addressed. Evolution of the available VLEs, such as the Blackboard and Brightspace used widely now in western medical schools might also emerge as competitors to such freely available social media-based learning platforms. Moreover, there is a lack of understanding regarding such survey tools offered by the different social media portals, which needs further exploration before the uses of such platforms can be integrated into mainstream anatomy education.

## Take Home Messages

• The survey tool of Twitter, as a Web 2.0 technology, is providing novel opportunities for developing multiple-choice practice questions (MCQs) as a formative assessment strategy.
• A fourteen weeks-long study was undertaken under strict anonymity and entirely voluntary participation, where one MCQ was tweeted each working day while the correct answer to the question revealed the next day.
• The student engagement with the surveys, as interpreted from the numerical parameters extracted from the data analytics suite inbuilt on Twitter, overall improved throughout the study.
• The student feedback regarding the exercise was positive and encouraging.
• Such an assessment strategy can endorse learning of anatomy if delivered in a timely and integrated fashion in alignment with the learning objectives.

## Notes On Contributors

SOURAV BHATTACHARJEE, M.B.B.S., Ph.D., is an assistant professor of anatomy in the Section of Veterinary Biosciences at University College Dublin (UCD) School of Veterinary Medicine in Dublin, Ireland. He teaches both human and veterinary anatomy with a focus on the abdominal and pelvic anatomy. He is a member of the Anatomical Society (AS) while he was also involved in teaching anatomy in the Imperial College London (UK). He is interested in the uses of emerging platforms, such as social media and imaging modalities in anatomy education. ORCID: https://orcid.org/0000-0002-6528-6877.
