# Supplementary material for: #anatomymcq—A pilot study on using the Twitter survey tool as a formative assessment strategy
Source: MedEdPublish (2016). 2020 Jun 30;9:141. [Version 1] doi: 10.15694/mep.2020.000141.1 (PMC10702688; doi:10.15694/mep.2020.000141.1)
Supplement: Peer review history

# Reviewer report: Reviewer response for version 1

**DOI:** 10.21956/mep.20055.r30751

**Published:** 2020-08-06

**Authors:** Hosam Eldeen Gasmalla

**Affiliations:** 1 Sudan International University

This review has been migrated. The reviewer awarded 4 stars out of 5 This is an interesting article, however, the twitter survey tool is not helpful if we intend to analyze the MCQs itself in terms of item difficulty or item discrimination owing to its anonymity, thus it can only be confined to formative assessment, in the other hand, there are no scores, feedback is a cornerstone concept in formative assessment, part of the feedback is by giving the correct answer to the student, also part of the feedback is to collect information from the scores obtained by the student, this information will help the teacher to identify weaknesses and to provide tailored formative assessment for each student or group of students, this follow-up process seems to be difficult using tweeter, again due to its anonymity. I'm curious, is the engagement rate a precise way to determine the actual number of participants? because it also includes those who retweeted, can a person retweet a poll without answering it, the engagement includes beside the retweets, the replies and follows, they show the growing interest but do they show the actual participants? I recommend they relying on the number of votes as an indicator of the actual interest. The selection of twitter or Facebook depends on the most trending platform for the students, in some countries, Facebook is dominant, while in other countries the opposite can be seen.Overall, this is a very interesting article, it waives the way of similar research to be conducted in order to explore this new approach.

Reviewer Expertise:

NA

No decision status is available

# Reviewer report: Reviewer response for version 1

**DOI:** 10.21956/mep.20055.r30752

**Published:** 2020-08-04

**Authors:** Keith Wilson

**Affiliations:** 1 Dalhousie University

This review has been migrated. The reviewer awarded 4 stars out of 5 The present study was designed to explore a low-cost social media platform, Twitter, in the provision of formative anatomy assessment at a veterinary school in Ireland. The study was conducted over a 14-week period and allowed for the observation of sustained engagement in such a tool. Using a technology such as Twitter allowed the author to collect a number of engagement metrics that helped support the conclusions. It also allowed for anonymity of its users. The author highlighted the limitations of the tool as it stood in 2017 yet postulated potential future use and enhancements (including not being able to use images).The author highlights that Twitter may not be a ‘disruptive technology’ for anatomy education at this time however increasing use may afford new opportunities. I would be curious to know how many students used Twitter in general and how the students performed on the surveys (e.g. overall how many got the formative questions correct and whether there were differences between anatomy components). The author sought feedback from the students during the course of the study (which showed student enthusiasm) and although unlikely, it had the potential to influence their responses on feedback questionnaires. The author really presents an opportunity to explore a rich dataset using tools available on a social media platform – this could inform much future research.The part of this study that I am particularly excited about is using a tool such as Twitter to help students engage in ‘spaced learning’ and using the ‘testing effect’ to enhance learning. The timeliness of this study, particularly in light of the present COVID-19 crisis, highlights alternative ways to expand beyond the walls of health professions education. Any educator who has an interest in the integration of social media into curricula should read this article. It is well written and contains numerous key points that could be applied to numerous settings.

Reviewer Expertise:

NA

No decision status is available

# Reviewer report: Reviewer response for version 1

**DOI:** 10.21956/mep.20055.r30755

**Published:** 2020-07-05

**Authors:** Mohamed Hany Shehata

**Affiliations:** 1 Arabian Gulf University

This review has been migrated. The reviewer awarded 4 stars out of 5 Well-written article that adds to the area of using social media in medical education. Linking the used tool to student performance might be an important objective evaluation to the experience.

Reviewer Expertise:

NA

No decision status is available

# Reviewer report: Reviewer response for version 1

**DOI:** 10.21956/mep.20055.r30753

**Published:** 2020-07-04

**Authors:** P Ravi Shankar

**Affiliations:** 1 American International Medical University

This review has been migrated. The reviewer awarded 4 stars out of 5 The author explores the use of Twitter as a tool for teaching-learning and formative assessment in a health science school in Ireland. The article is well-written and placed within the proper theoretical framework. The strengths and limitations of the study have been well brought out. There have been other studies mentioning that students may prefer ‘open’ platforms like Twitter, and Facebook compared to interacting through institutional learning platforms as the author has mentioned. There has been some delay between conduct of the study and publication of the results of the study.The author has mentioned the limitations of conducting surveys on Twitter compared to Facebook and has mentioned that Facebook offers more functionality. Considering this why did the author choose Twitter as the platform for the MCQs? The article is a useful addition to the literature on the use of social media in medical education.

Reviewer Expertise:

NA

No decision status is available

# Reviewer report: Reviewer response for version 1

**DOI:** 10.21956/mep.20055.r30754

**Published:** 2020-07-01

**Authors:** Felix Silwimba

**Affiliations:** 1 University of Lusaka

This review has been migrated. The reviewer awarded 5 stars out of 5 this is a very informative study, challenging and encouraging as lecture to know more about SOME us and benefits in medical education.

Reviewer Expertise:

NA

No decision status is available
